# Author Correction: Linking diet switching to reproductive performance across populations of two critically endangered mammalian herbivores

**DOI:** 10.1038/s42003-024-06167-9

**Published:** 2024-04-26

**Authors:** Nick Harvey Sky, Jake Britnell, Rachael Antwis, Tyler Kartzinel, Daniel Rubenstein, Phil Toye, Benedict Karani, Regina Njeru, Danielle Hinchcliffe, Jamie Gaymer, Samuel Mutisya, Susanne Shultz

**Affiliations:** 1 Department of Earth and Environmental Sciences, University of Manchester, Manchester, M13 9NT UK; 2 North of England Zoological Society, Chester Zoo, Upton-by-Chester, CH2 1LH UK; 3 School of Environment and Life Sciences, University of Salford, Salford, M5 4WX UK; 4 Department of Ecology, Evolution, and Organismal Biology, Brown University, 85 Waterman Street, Providence, RI 02912 USA; 5 Institute at Brown for Environment and Society, Brown University, 85 Waterman Street, Providence, RI 02912 USA; 6 Department of Ecology and Evolutionary Biology, Princeton University, Princeton, NJ 08544-2016 USA; 7 International Livestock Research Institute and Centre for Tropical Livestock Genetics and Health, Nairobi, P.O. Box 30709-00100 Kenya; 8 School of Biological and Environmental Sciences, Liverpool John Moores University, Liverpool, L3 3AF UK; 9 Ol Jogi Ltd., PO Box 259-10400, Nanyuki, Kenya; 10 Ol Pejeta Conservancy, PO Box 167, Nanyuki, Kenya

**Keywords:** Molecular ecology, Conservation biology, Animal behaviour

Correction to: *Communications Biology* 10.1038/s42003-024-05983-3, published online 15 March 2024

In the original version of the article, Danielle Hinchcliffe was missing from the author list. This has now been corrected and the full author list is:

Nick Harvey Sky^1,2^, Jake Britnell^1,2^, Rachael Antwis^3^, Tyler Kartzinel^4,5^, Daniel Rubenstein^6^, Phil Toye^7^, Benedict Karani^7^, Regina Njeru^7^, Danielle Hinchcliffe^8^, Jamie Gaymer^9^, Samuel Mutisya^10^ & Susanne Shultz^1,2^

The affiliation of Danielle Hinchcliffe has also been added:

^8^School of Biological and Environmental Sciences, Liverpool John Moores University, Liverpool, L3 3AF, UK

The “Author contributions” section has been modified to read:

N.H.S. contributed to conceptualisation, methodology, formal analysis, investigation, data curation, writing—original draft, writing—review & editing, visualisation, project administration and funding acquisition. J.A. contributed to methodology, formal analysis, investigation, data curation, writing—original draft, writing—review & editing and visualisation. R.A. contributed to methodology, formal analysis, resources and writing—review & editing. T.R.K. contributed to methodology, formal analysis, and writing—review & editing. D.I.R. contributed to formal analysis, data curation, and writing—review & editing. P.T. contributed to resources, writing—review & editing and project administration. B.E.K. contributed to investigation, resources and writing—review & editing. R.N. contributed to investigation, resources and writing—review & editing. D.H. contributed to investigation, data curation, and writing—review & editing. J.G. contributed to methodology, resources and data curation. S.M. contributed to methodology, resources and data curation. S.S. contributed to conceptualisation, methodology, formal analysis, investigation, writing—original draft, writing—review & editing, visualisation, supervision, project administration and funding acquisition.

