# Supplementary material for: Linking diet switching to reproductive performance across populations of two critically endangered mammalian herbivores
Source: Commun Biol. 2024 Mar 15;7:333. doi: 10.1038/s42003-024-05983-3 (PMC10943211; doi:10.1038/s42003-024-05983-3)
Supplement: Supplementary file 2 — Supplementary Information [file 42003_2024_5983_MOESM2_ESM.pdf]

Linking diet switching to reproductive performance across populations of two Critically Endangered mammalian herbivores - Supplementary Information

Results

Supplementary Table 1) perMANOVA with 10,000 permutations for weighted Unifrac dissimilarity in diet composition across ecological gradient varying with environmental variables. NDVI denotes normalised difference vegetation index as a proxy of grass availability and forage. Variable = predictors in the model, Df = degrees of freedom, SS= Sum of squares, R^2=percentage of variance explained by the predictor, Pseudo-F = pseudo-F value, p = p valuers

**Species**		**Variable**	**Df (explanatory variable, total)**	**SS**	R^2	**Pseudo-F**	**p**
**Black Rhino**	Model 1	Population	2,213	0.072	0.21	30.22	≤0.0001***
		Sampling period	1,213	0.020	0.059	16.85	≤0.0001***
**Grevy's Zebra**	Model 1	Population	2,153	0.23	0.66	84.68	≤0.0001***
		Sampling period	1,153	0.019	0.054	27.67	≤0.0001***

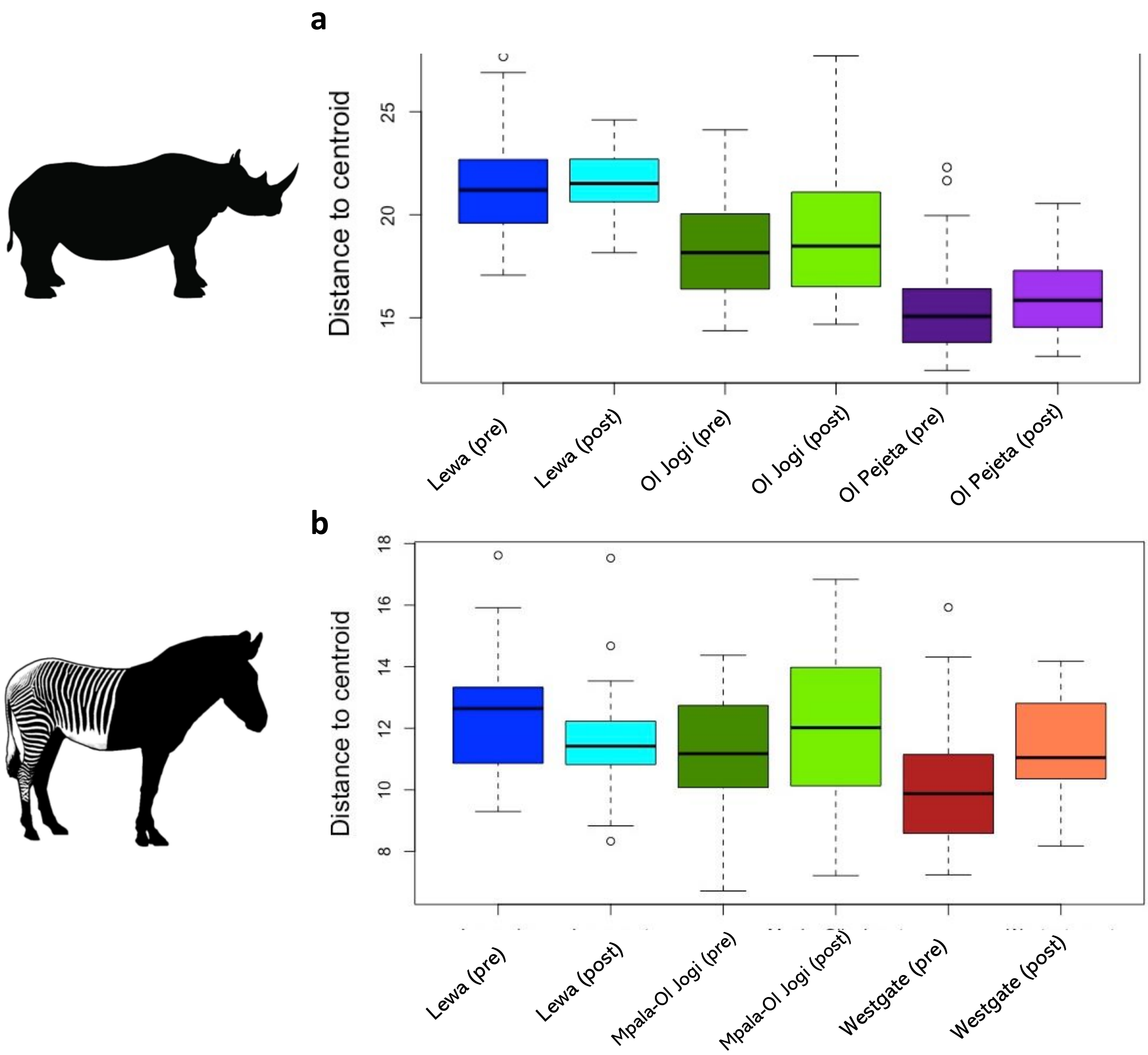

Supplementary Figure 1) Beta dispersion of diet dietary diversity for a) black rhino (n=214) and b) Grevy's zebra (n=154). The lower, middle and upper horizontal lines show the 25th percentile (lower hinge), mean and 75th percentile (upper hinge) respectively. Whiskers extend to the largest and smallest values no further than 1.5 times the inter-quartile range away from the hinges. Data points beyond the whiskers are shown as points.

Supplementary Table 2) Tukey test table for beta dispersion within reserve across seasons (pre- and post-rains) for black rhino (n=214) and Grevy's zebra (n=154)

Species	Comparison	*Difference*	*Lower bound*	*Upper bound*	*p*
**Black Rhino**	Ol Jogi-Lewa	-2.30	-3.19	-1.41	<0.001***
	Ol Pejeta-Lewa	-5.87	-6.92	-4.82	<0.001***
	Ol Pejeta-Ol Jogi	-3.56	-4.54	-2.59	<0.001***
**Grevy's Zebra**	Mpala-Ol Jogi – Lewa	-0.32	-1.27	0.63	0.70
	Westgate – Lewa	-1.08	-2.14	-0.021	0.044*
	Westgate – Mpala-Ol Jogi	-0.76	-1.73	0.21	0.15

Supplementary Table 3) Table of Spearman's rank correlations between major black rhino dietary plant family groups across populations. We applied a Hom correction to the nine tests presented here and the three tests presented in the main text together. Degrees of freedom presented in brackets with t statistic.

	**Lewa (n=65)**	**Ol Jogi (n=97)**	**Ol Pejeta (n=50)**
**Fabaceae vs Ebenaceae**	r=-0.48, CI = -0.65 - -0.27, t(65) = -4.42, p<0.001***	r=-0.50, CI = -0.64 - -0.33, t(95) = -5.62, p<0.001***	r=-0.49, CI = -0.68 - -0.25, t(48) = -3.93, p=0.0021**
**Fabaceae vs Poaceae**	r=-0.24, CI = -0.46 - -0.00, t(65) = -2.01, p=0.28	r=-0.01, CI = -0.21 - 0.19, t(95) = -0.11, p=0.92	r=-0.38, CI = -0.59, -0.11, t(48) = -2.84, p=0.046*
**Ebenaceae vs Poaceae**	r=-0.21, CI = -0.43 - 0.04, t(65) = -1.70, p=0.28	r=-0.13, CI = -0.32 - 0.07, t(95) = -1.25, p=0.43	r=-0.28, CI = -0.52, -0.00, t(48) = -2.04, p=0.28

Discussion

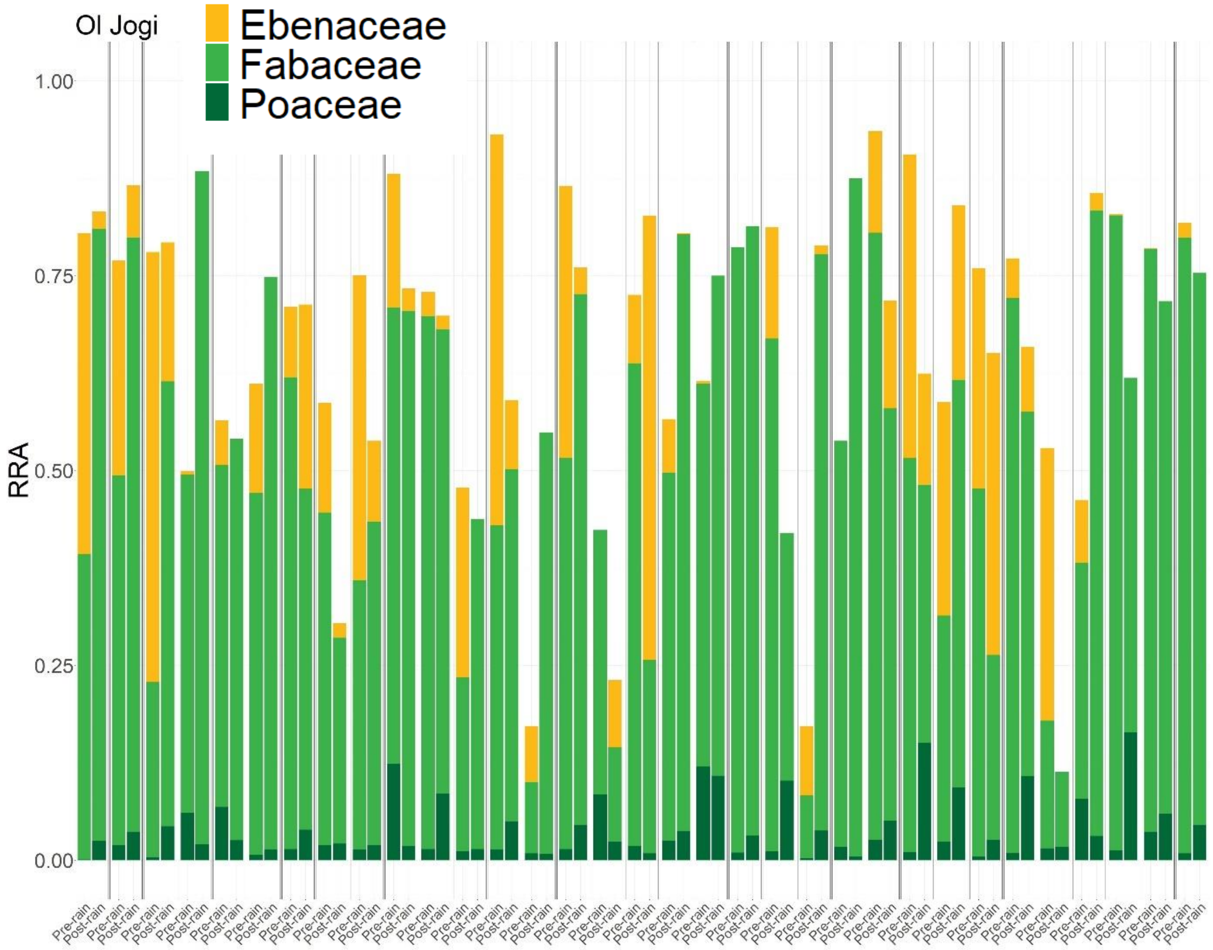

Supplementary Figure 2) Black Rhino individual diet composition at family level within Ol Jogi split by season. Only the three main study families are presented.

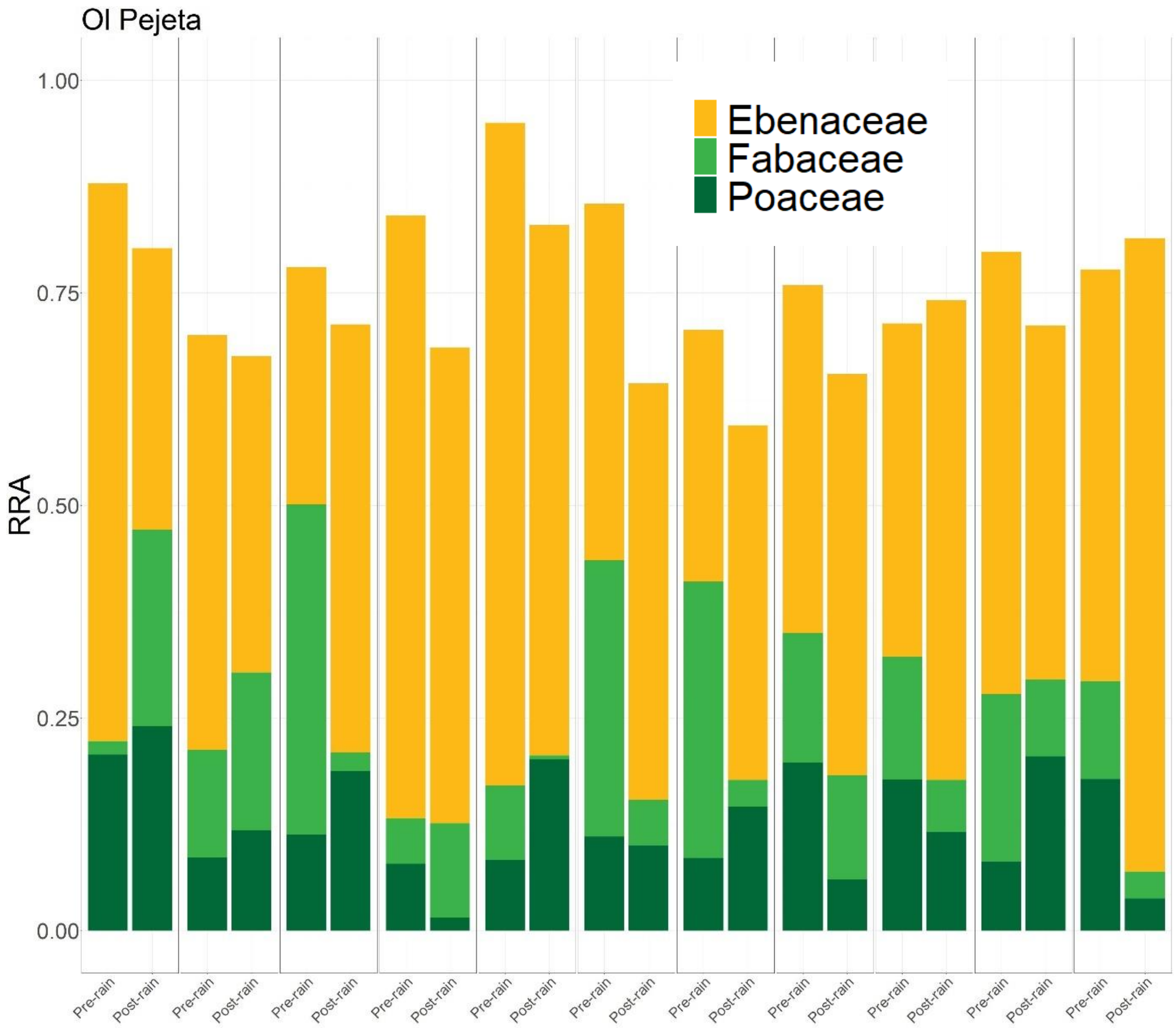

Supplementary Figure 3) Black Rhino individual diet composition at family level within Ol Pejeta split by season. Only the three main study families are presented.

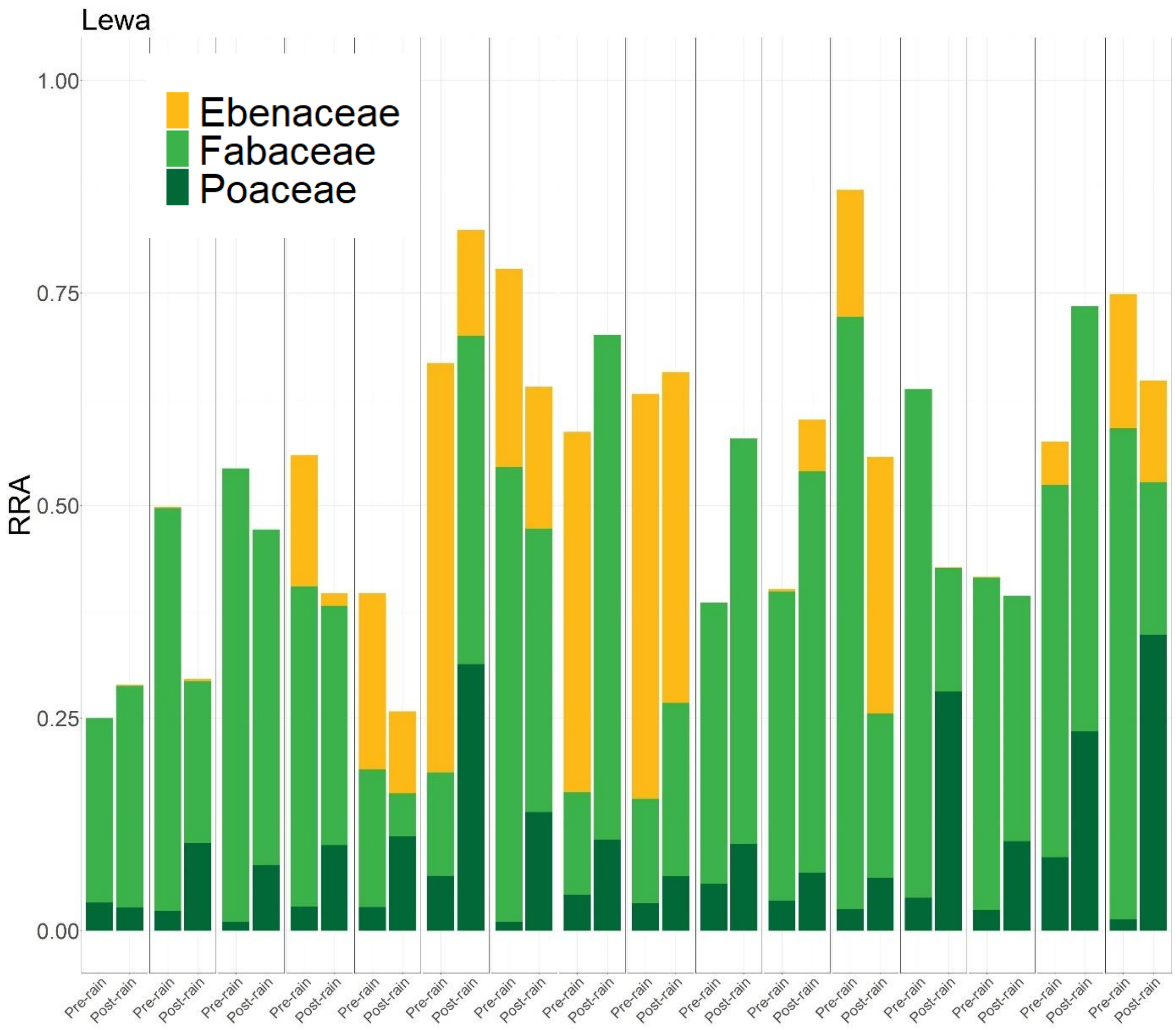

Supplementary Figure 4) Black Rhino individual diet composition at family level within Lewa split by season. Only the three main study families are presented.

Supplementary Table 4) Microbiome and diet shifts across seasons in Grevy's zebra and Black Rhino, estimated as distance between dietary season centroid for both diet and microbiome

Species	Reserve	Microbiome shift	Diet shift
**Zebra**	Westgate	0.058	0.028
**Zebra**	Lewa	0.020	0.027
**Zebra**	Mpala -Ol jogi	0.022	0.015
**Rhino**	Ol Pejeta	0.031	0.025
**Rhino**	Lewa	0.085	0.059
**Rhino**	Ol Jogi	0.047	0.10

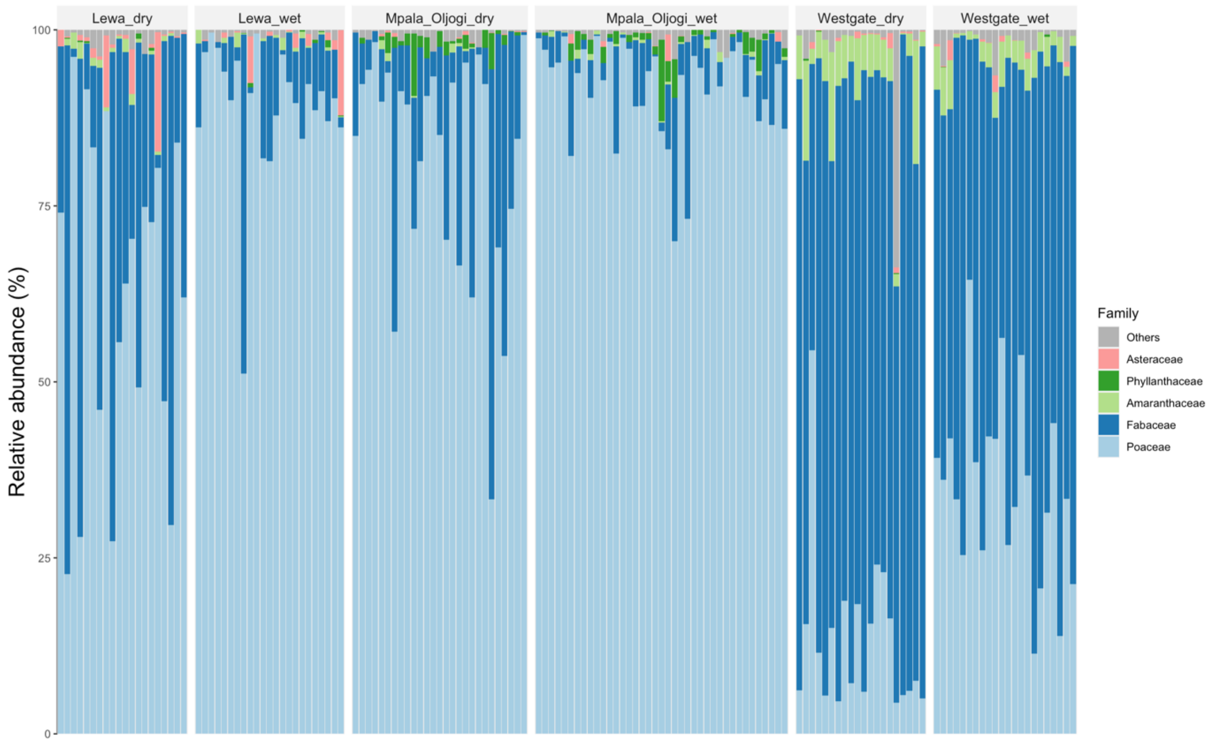

Supplementary Figure 5) Grevy's zebra diet composition across geographic areas split by reserve and season. Analysed at Family level

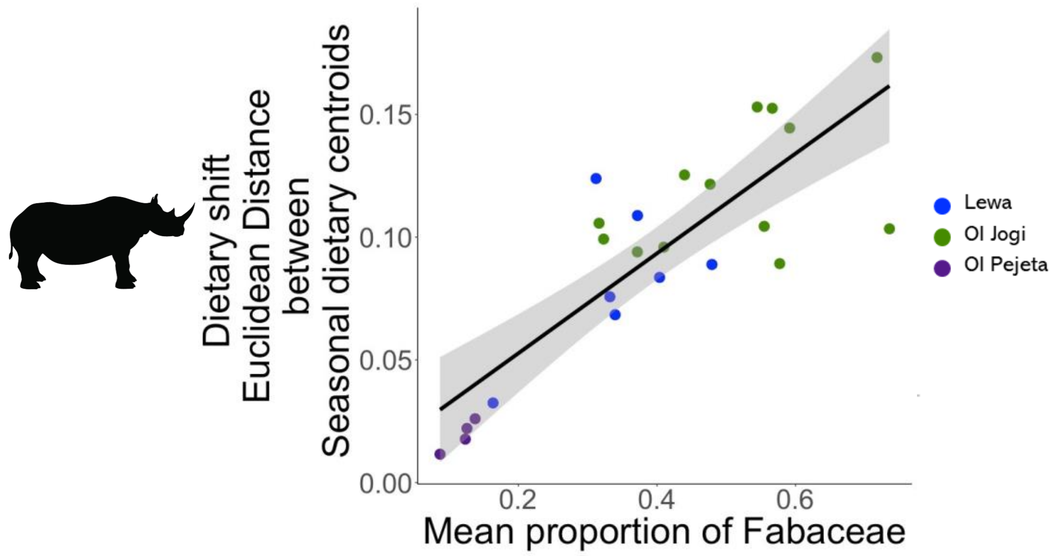

Supplementary Figure 6) Dietary shifts in female black rhino which have bred at least once are associated with increased mean proportion of Fabaceae in the diet pre and post rains. The more Fabaceae black rhino consume across pre and post rains the greater the dietary shift across seasons.

Supplementary Table 5) Output of a linear model testing whether individual dietary shifts (measured by the Euclidean distance between the centroids of ordinations using clr-transformed RRAs) correlate with mean individual-level RRAs of the three plant families in female black rhino which bred at least once. Black rhino dietary shift in females of breeding age is significantly predicted by the amount of Fabaceae in black rhino diets, but not by the other two plant families. This shows that black rhino change their diets more when they eat more Fabaceae. Model statistics: $R^2 = 0.66$, $F=16.2_{3,20}$, $p<0.001$

**Model**	**AIC**	**Variable**	β	**se**	**t**	**p**
**Dietary shift vs key dietary plant families**	-101.52	Legumes	0.17	0.046	3.66	0.0016**
		Grasses	0.0024	0.088	0.027	0.98
		Ebenaceae	-0.044	0.041	-1.07	0.30

Methods

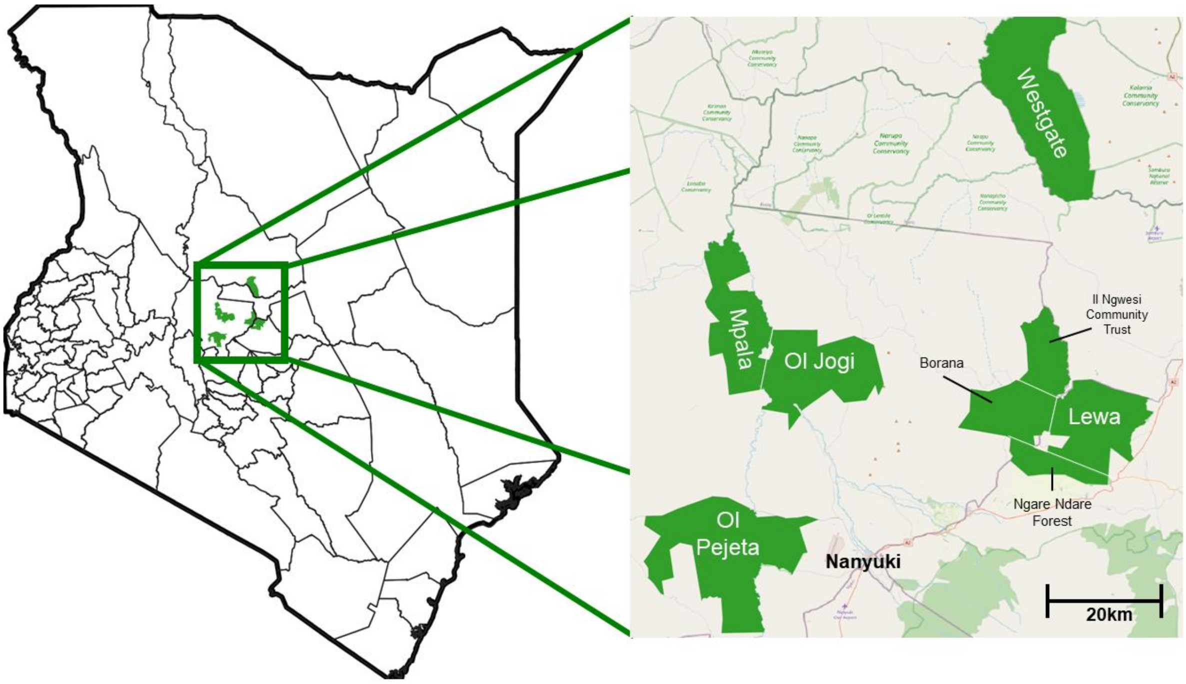

Supplementary Figure 7) Map of the placement of the study reserves within Kenya: Ol Pejeta Conservancy (black rhino), Ol Jogi Conservancy (black rhino and Grevy's zebra), Lewa Wildlife Conservancy (black rhino and Grevy's zebra), Mpala Ranch Research Centre (Grevy's zebra) and Westgate Community Conservancy (Grevy's zebra). Lewa, Ol Jogi and Ol Pejeta have fence gaps that allow all animals apart from black and white rhino to migrate in and out of them. The fence between Lewa and neighbouring Borana conservancy was removed in 2015, and black rhino on Lewa can move in and out of Ngare Ndare Forest, and Borana.

Supplementary Table 6) Amplicon primers

16S rRNA	
Forward	GTGCCAGCMGCCGCGGTAA
Reverse	GGACTACHVGGGTWTCTAAT
***trn*L(UAA)**	
Forward - *trn*L(UAA)g	GGGCAATCCTGAGCCAA
Reverse – *trn*L(UAA)h	CCATTGAGTCTCTGCACCTATC

Supplementary Table 7) Diet index primers

**Forward**	
N501_i5	TAGATCGC
N502_i5	CTCTCTAT
N503_i5	TATCCTCT
N504_i5	AGAGTAGA
N505_i5	GTAAGGAG
N506_i5	ACTGCATA
N507_i5	AAGGAGTA
N508_i5	CTAAGCCT
N510_i5	CGTCTAAT
N511_i5	TCTCTCCG
N513_i5	TCGACTAG
N515_i5	TTCTAGCT
N516_i5	CCTAGAGT
N517_i5	GCGTAAGA
N518_i5	CTATTAAG
N520_i5	AAGGCTAT
**Reverse**	
N701_i7	TCGCCTTA
N702_i7	CTAGTACG
N703_i7	TTCTGCCT
N704_i7	GCTCAGGA
N705_i7	AGGAGTCC
N706_i7	CATGCCTA
N707_i7	GTAGAGAG
N708_i7	CCTCTCTG
N709_i7	AGCGTAGC
N710_i7	CAGCCTCG
N711_i7	TGCCTCTT
N712_i7	TCCTCTAC
N714_i7	TCATGAGC
N715_i7	CCTGAGAT
N716_i7	TAGCGAGT
N718_i7	GTAGCTCC
N719_i7	TACTACGC
N720_i7	AGGCTCCG
N721_i7	GCAGCGTA
N722_i7	CTGCGCAT
N723_i7	GAGCGCTA
N724_i7	CGCTCAGT

N726_i7	GTCTTAGG
N727_i7	ACTGATCG

Supplementary Table 8) Bacteria index primers

**Forward**	
SA501_i5	ATCGTACG
SA502_i5	ACTATCTG
SA503_i5	TAGCGAGT
SA504_i5	CTGCGTGT
SA505_i5	TCATCGAG
SA506_i5	CGTGAGTG
SA507_i5	GGATATCT
SA508_i5	GACACCGT
SB501_i5	CTACTATA
SB502_i5	CGTTACTA
SB503_i5	AGAGTCAC
SB504_i5	TACGAGAC
SB505_i5	ACGTCTCG
SB506_i5	TCGACGAG
SB507_i5	GATCGTGT
SB508_i5	GTCAGATA
**Reverse**	
SA701_i7	CGAGAGTT
SA702_i7	GACATAGT
SA703_i7	ACGCTACT
SA704_i7	ACTCACTG
SA705_i7	TGAGTACG
SA706_i7	CTGCGTAG
SA707_i7	TAGTCTCC
SA708_i7	CGAGCGAC
SA709_i7	ACTACGAC
SA710_i7	GTCTGCTA
SA711_i7	GTCTATGA
SA712_i7	TATAGCGA
SB701_i7	CTCGACTT
SB702_i7	CGAAGTAT
SB703_i7	TAGCAGCT
SB704_i7	TCTCTATG
SB705_i7	GATCTACG
SB706_i7	GTAACGAG
SB707_i7	ACGTGCGC
SB708_i7	ATAGTACC
SB709_i7	GCGTATAC
SB710_i7	TGCTCGTA
SB711_i7	AACGCTGA
SB712_i7	CGTAGCGA

Supplementary Table 9) Number of reads at each stage of bioinformatic processing for black rhino diet

Stage	Total	Mean	Range
Raw	1433519	6371.196	4-254516
Filtered	1422397	6321.764	3-252140
Denoised Forward	1418972	6306.542	2-251863
Denoised Reverse	1419375	6308.333	2-251946
Merged	1409166	6262.96	0-250384
Chimeras removed	1405616	6247.182	0 -247284
12 Samples below 1000 reads removed	1153391	5414.981	1446-206593

Supplementary Table 10) Number of ASVs at each stage of bioinformatic processing for black rhino diet. After processing, 139 ASVs had sequence lengths under 50 bases. These remained unassigned to taxa apart from 5 Fabaceae and 2 Poaceae reference sequences which were assigned manually using exact sequence matches.

Stage	Number of ASVs	Length range	Modal length
Before 12 samples with below 1000 reads removed	349	26 – 115	51
After samples with below 1000 reads removed	349	26 – 115	51

Supplementary Table 11) Number of reads at each stage of bioinformatic processing for Grevy's zebra diet

Stage	Total	Mean	Range
Raw	1040514	6585.525	19 -12123
Filtered	1022439	6471.133	16 -12069
Denoised Forward	1020251	6457.285	14 -12063
Denoised Reverse	1020635	6459.715	11 -11992
Merged	1012797	6410.108	0 – 11932
Chimeras removed	1011612	6402.608	0 – 11917
4 Samples below 1000 reads removed	918871	5966.695	1072 – 11820

Supplementary Table 12) Number of ASVs at each stage of bioinformatic processing for Grevy's zebra diet. After processing, 142 ASVs had sequence lengths under 50 bases. These remained unassigned to taxa apart from 5 Fabaceae and 2 Poaceae reference sequences which were assigned manually using exact sequence matches.

Stage	Number of ASVs	Length range	Modal length
Before 4 samples with below 1000 reads removed	472	21 – 152	52
After 4 samples with below 1000 reads removed	471	23 – 152	52

Supplementary Table 13) Number of reads at each stage of bioinformatic processing for black rhino microbiome

Stage	Total	Mean	Range
Raw	4608550	20391.81	179 – 114486
Filtered	2819815	12477.06	55 – 79335
Denoised Forward	2542378	11249.46	6 – 77653
Denoised Reverse	2583221	11430.18	2 – 78142
Merged	2256748	9985.611	0 – 74349
Chimeras removed	2229361	9864.429	0 – 71907
Sequences outside expected range removed	2229163	9863.553	0 – 71907
27 Samples below 2000 reads removed	2199901	11054.78	2012 – 71907
Sequences in negative control removed	2199723	11053.88	2012 – 71907

Supplementary Table 14) Number of ASVs at each stage of bioinformatic processing for black rhino microbiome

Stage	Number of ASVs	Length range	Modal length
Before ASVS outside 250-260 removed	7309	200-268	253
After ASVS outside 250-260 removed	7294	250 – 259	254
After 27 samples with below 2000 reads removed	7173	250 – 258	253
After negative control sequences removed	7172	250 – 258	253

Supplementary Table 15) Number of reads at each stage of bioinformatic processing for Grevy's zebra microbiome

Stage	Total	Mean	Range
Raw	7573950	47936.39	132 – 1067479
Filtered	4694606	29712.7	14 – 726368
Denoised Forward	4234967	26803.59	1 – 706493
Denoised Reverse	4322173	27355.53	8 – 708389
Merged	3752821	23752.03	0 – 674246
Chimeras removed	3600261	22786.46	0 – 636331
Sequences outside expected range removed	3600256	22786.43	0 – 636331
2 Samples below 2000 reads removed	3598960	23070.26	28476 – 636331
Sequences in negative control removed	3598960	23070.26	2846 – 636331

Supplementary Table 16) Number of ASVs at each stage of bioinformatic processing for Grevy's zebra microbiome

Stage	Number of ASVs	Length range	Modal length
Before ASVS outside 250-260 removed	8750	241 – 268	253
After ASVS outside 250-260 removed	8748	251 – 255	254
After 2 samples with below 2000 reads removed	8746	251 – 2558	253
After negative control sequences removed	8746	250 – 258	253

Supplementary Table 17) Percentage of ASVs assigned to genus and family level for diet

Level	Black rhino	Grevy's zebra
Genus	39%	36%
Family	57%	61%

Supplementary Table 18) Percentage of ASVs assigned to genus and family level for microbiome

Level	Black rhino	Grevy's zebra
Genus	50%	47%
Family	72%	81%

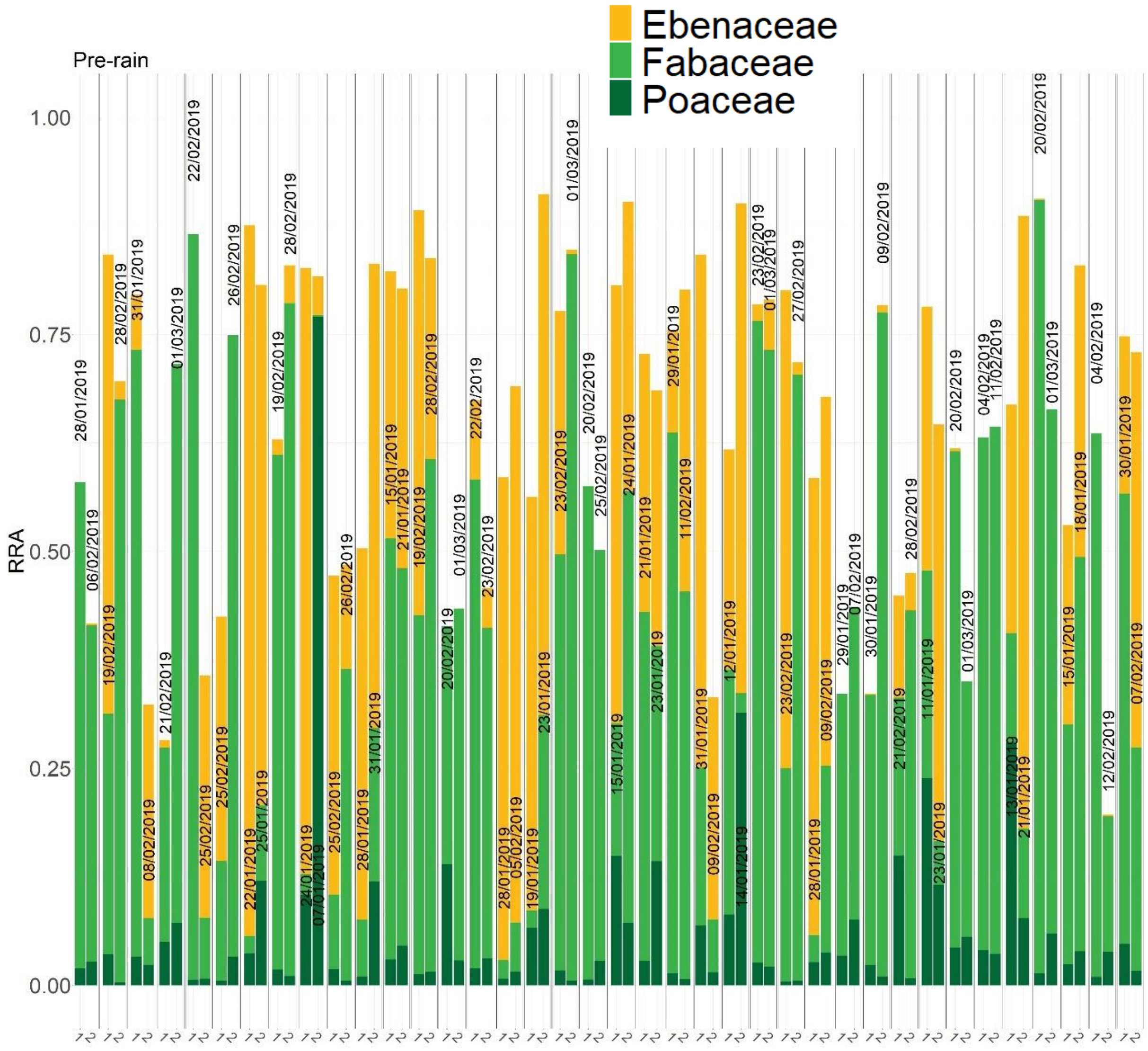

Supplementary Figure 8) Black Rhino individual diet composition at family level for individuals which were sampled more than once in the pre-rains season. On the x-axis, 1 indicates the first sample taken from that individual in that season, and 2 indicates the second. The date each sample was taken is also presented. There is no consistent pattern of variation between early and late in the pre-rain season. Only the three main study families are presented.

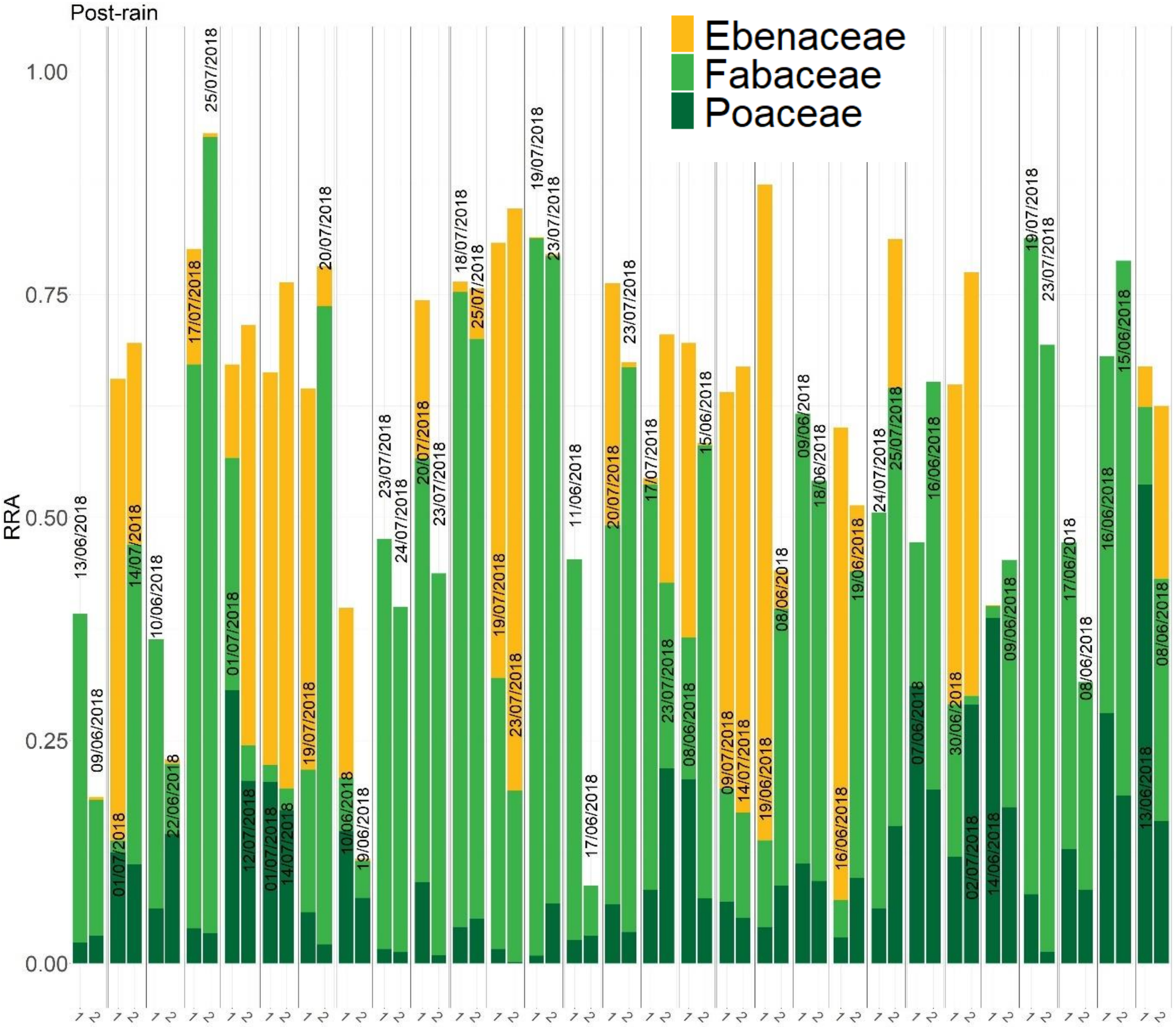

Supplementary Figure 9) Black Rhino individual diet composition at family level for individuals which were sampled more than once in the post-rains season. On the x-axis, 1 indicates the first sample taken from that individual in that season, and 2 indicates the second. The date each sample was taken is also presented. There is no consistent pattern of variation between early and late in the post-rain season. Only the three main study families are presented.
