# Supplementary material for: Linking diet switching to reproductive performance across populations of two critically endangered mammalian herbivores
Source: Commun Biol. 2024 Mar 15;7:333. doi: 10.1038/s42003-024-05983-3 (PMC10943211; doi:10.1038/s42003-024-05983-3)
Supplement: Supplementary file 3 — Description of Additional Supplementary Files [file 42003_2024_5983_MOESM3_ESM.pdf]

Description of Additional Supplementary Files

File name: Supplementary Data 1
Description: Relative abundances of the twenty plant ASVs at the genus level with the highest mean relative abundance across reserves and seasons in black rhino.

File name: Supplementary Data 2
Description: Relative abundances of the twenty plant ASVs at the genus level with highest mean relative abundance across reserves and seasons in Grevy's zebra.

File name: Supplementary Data 3
Description: Relative abundances of the twenty bacteria ASVs at the family level with the highest mean relative abundance across reserves and seasons in black rhino.

File name: Supplementary Data 4
Description: Relative abundances of twenty bacteria ASVs at the family level with the highest mean relative abundance across reserves and seasons in Grevy's zebra.

File name: Supplementary Data 5
Description: Loading scores for principal component 1 of black rhino for bacterial families with scores > 0.3 or <-0.3 Positive loading scores indicate bacterial families which were associated with increasing Fabaceae in the diet.

File name: Supplementary Data 6
Description: Loading scores for principal component 2 of black rhino for bacterial families with scores > 0.3 or <-0.3. Positive loading scores indicate bacterial families which were associated with increasing Fabaceae in the diet.

File name: Supplementary Data 7
Description: Loading scores for principal component 1 of Grevy's zebra for bacterial families with scores > 0.3 or <-0.3. Positive loading scores indicate bacterial families which were associated with increasing Fabaceae in the diet.

File name: Supplementary Data 8
Description: Loading scores for principal component 2 of Grevy's zebra for bacterial families with scores > 0.3 or <-0.3. PC2 was not associated with NDVI, Poaceae or Fabaceae variation along this axis is likely driven by other factors not accounted for in this study.

File name: Supplementary Data 9
Description: Source data for all figures that present results.
