# Supplementary material for: Linking diet switching to reproductive performance across populations of two critically endangered mammalian herbivores
Source: Commun Biol. 2024 Mar 15;7:333. doi: 10.1038/s42003-024-05983-3 (PMC10943211; doi:10.1038/s42003-024-05983-3)
Supplement: Supplementary file 6 — Reporting Summary [file 42003_2024_5983_MOESM6_ESM.pdf]

nature portfolio

Corresponding author(s): Nick Harvey Sky

Last updated by author(s): Jan 31, 2024

Reporting Summary

Nature Portfolio wishes to improve the reproducibility of the work that we publish. This form provides structure for consistency and transparency in reporting. For further information on Nature Portfolio policies, see our Editorial Policies and the Editorial Policy Checklist.

Statistics

For all statistical analyses, confirm that the following items are present in the figure legend, table legend, main text, or Methods section.

n/a	Confirmed	
☐	☒	The exact sample size (*n*) for each experimental group/condition, given as a discrete number and unit of measurement
☐	☒	A statement on whether measurements were taken from distinct samples or whether the same sample was measured repeatedly
☐	☒	The statistical test(s) used AND whether they are one- or two-sided<br>*Only common tests should be described solely by name; describe more complex techniques in the Methods section.*
☐	☒	A description of all covariates tested
☐	☒	A description of any assumptions or corrections, such as tests of normality and adjustment for multiple comparisons
☐	☒	A full description of the statistical parameters including central tendency (e.g. means) or other basic estimates (e.g. regression coefficient) AND variation (e.g. standard deviation) or associated estimates of uncertainty (e.g. confidence intervals)
☐	☒	For null hypothesis testing, the test statistic (e.g. *F*, *t*, *r*) with confidence intervals, effect sizes, degrees of freedom and *P* value noted<br>*Give P values as exact values whenever suitable.*
☒	☐	For Bayesian analysis, information on the choice of priors and Markov chain Monte Carlo settings
☒	☐	For hierarchical and complex designs, identification of the appropriate level for tests and full reporting of outcomes
☐	☒	Estimates of effect sizes (e.g. Cohen's *d*, Pearson's *r*), indicating how they were calculated

Our web collection on statistics for biologists contains articles on many of the points above.

Software and code

Policy information about availability of computer code

Data collection	We used Illumina BaseSpace to collect sequences from the MiSeq and download them as fasta files for further processing.
Data analysis	All software used for data analysis is open source and freely available. All geographical analyses were conducted in QGIS version 3.16. We used Cutadapt 2.1 to remove the forward and reverse adapters present in the diet MiSeq reads. The rest of the processing and analysis was conducted in Rstudio for R (v4.2.0). All packages used for both bioinformatic processing and statistical analysis are described in the methods and cited in full.

For manuscripts utilizing custom algorithms or software that are central to the research but not yet described in published literature, software must be made available to editors and reviewers. We strongly encourage code deposition in a community repository (e.g. GitHub). See the Nature Portfolio guidelines for submitting code & software for further information.

Data

Policy information about availability of data

All manuscripts must include a data availability statement. This statement should provide the following information, where applicable:

- Accession codes, unique identifiers, or web links for publicly available datasets
- A description of any restrictions on data availability
- For clinical datasets or third party data, please ensure that the statement adheres to our policy

The raw metabarcoding output fasta files, and some processed metabarcoding and sample data in xslx files can accessed on Zenodo zenodo.org - Linking diet

switching to reproductive performance across populations of two Critically Endangered mammalian herbivores 10.5281/zenodo.10575034

Data regarding Kenyan black rhino and Grevy's zebra are treated as sensitive and confidential. There are therefore restrictions on the data that we can make available. Due to these confidentiality considerations, the sample data stored on Zenodo do not include locations of sample collection within each reserve for either species, or the identity or breeding data for black rhino. The data also only include the final processed values for NDVI and rainfall. The remote sensing data are available from the repositories cited in the methods, but we cannot provide the shapefiles or other spatial data used to calculate the final values for each sample. Access to any of the da that are not accessible on Zenodo must be agreed by the individual study reserves. Readers must send data requests to the corresponding author and these will be passed on to reserve management teams. Please note that access to the data is likely to require research permits and a data use agreement.

Research involving human participants, their data, or biological material

Policy information about studies with human participants or human data. See also policy information about sex, gender (identity/presentation), and sexual orientation and race, ethnicity and racism.

Reporting on sex and gender	NA
Reporting on race, ethnicity, or other socially relevant groupings	NA
Population characteristics	NA
Recruitment	NA
Ethics oversight	NA

Note that full information on the approval of the study protocol must also be provided in the manuscript.

Field-specific reporting

Please select the one below that is the best fit for your research. If you are not sure, read the appropriate sections before making your selection.

☐ Life sciences ☐ Behavioural & social sciences ☒ Ecological, evolutionary & environmental sciences

For a reference copy of the document with all sections, see nature.com/documents/nr-reporting-summary-flat.pdf

Ecological, evolutionary & environmental sciences study design

All studies must disclose on these points even when the disclosure is negative.

Study description	We evaluated temporal diet switching and microbiome variation across populations of two savanna herbivore species with different foraging strategies; the eastern black rhino (Diceros bicornis michaeli) and Grevy's zebra (Equus grevyi). We extracted plant and bacterial DNA from dung samples and used DNA metabarcoding to characterise variations in diet and microbiome between several reserves in Laikipia and Meru Counties in Kenya. We developed predictions of seasonal dietary changes using Optimal Foraging Theory. We then evaluated our predictions of diet switching before and after rains in different populations and whether this impacted on breeding performance.
Research sample	The research sample consists of 88 individual eastern black rhino (Diceros bicornis michaeli) across three Kenyan reserves, Lewa, Ol Jogi and Ol Pejeta, and an unkown number of individual Grevy's zebra (Equus grevyi) across three Kenyan conservancies and a research centre. The study reserves were chosen because they occur across a regional climatic gradient, and experience a range of rainfall levels. This rainfall gradient gives rise to different levels of primary productivity, different plant communities, and therefore diets available to the study animals, which also impact on gut microbiome. There were intra-reserve differences in rainfall and primary productivity as well as inter-reserve ones. We included measures of rainfall and NDVI on an individual level by estimating black rhino home ranges, and by estimating the distance that Grevy's zebra can move during their gut retention time. This allowed us to analyse how diets and microbiome, as well as individual seasonal changes in both diet and microbiome, vary at an individual level across this climatic gradient, and how these impacts on breeding performance.
Sampling strategy	Dung samples were taken from as many individuals as possible during the 8 week sampling periods (Black rhino: June – July 2018 and 431 January – March 2019; Grevy's: July-August 2018 and January-February 2019). From the samples that were available, we selected 226 black rhino samples and 158 for Grevy's zebra for metabarcoding. These were selected to give a spread of individuals from each reserve, from different areas of each reserve and both male and females. As black rhino were individually identifiable, we attempted to include two samples from each individual in each sampling session. A representation of the variation between repeat samples are in figures Supplementary Figures 8 and 9. As Grevy's zebra were not individually identifiable, sampling was conducted right across each reserve during each period, with a new area sampled each day, to attempt to sample as many different individuals as possible.<br>The sample size of the breeding analysis was determined by the number of individual breeding age black rhino females that were sampled, and for the Grevy's zebra this was determined by the granularity of the percentage of infants data which exists at the

county level.

Data collection

NDVI and rainfall data was based on remote sensing and downloaded and processed by NHS and JB as described in the methods section.
Black rhino demographic data was collected by the monitoring teams on each reserve. This was cleaned, processed and converted to the measure of inter-calving interval that we used in the analysis by NHS. For Grevy's zebra, we used percentage of infants in each of Laikipia, Meru and Samburu counties from Rubenstein et al. (2018) as an estimate of female reproductive rates.
DNA extraction, amplification and sequencing (using a MiSeq) was conducted according to the methods described by NHS, JAB, RJ and BEK at ILRI in Nairobi, using guidance from RA, TRK and SS.
The output of the MiSeq was processed by NHS and JAB, using guidance from RA, TRK and SS. After sequencing and bioinformatic processing, we tested whether community composition, alpha diversity and beta diversity of diet and microbiome, as well as the magnitude in seasonal shift in diet as well, was affected by rainfall and NDVI. We converted the number of reads assigned to different plant and bacterial taxa to relative read abundance (RRA). After examining the most prevalent plant families in the diet, we used the RRA of Fabaceae, Poaceae and Ebenaceae to test diet switching, and whether their presence in the diet was affected by environmental changes in rainfall and NDVI. We only included Fabaceae and Poaceae in the zebra analyses as they made up almost the entirety of their diets. We then tested whether microbiome composition and dietary shifts depended on these RRAs, and whether these RRAs correlated with each other.
After processing we had the following sample sizes. Rhino diet: Lewa-67, Ol Jogi-97, Ol Pejeta-50. Rhino microbiome: Lewa-59, Ol Jogi-92, Ol Pejeta-48. Zebra diet: Lewa-45, Westgate-42:, Mpala-Ol Jogi (also includes Karisia samples)-67. Zebra microbiome: Lewa-45, Westgate-43:, Mpala-Ol Jogi (also includes Karisia samples)-67.
Seasonal dietary shift was compared to individual female breeding performance in black rhino, and a RRA of dietary grass was compared to a measure of population performance in Grevy's zebra. The rhino breeding analysis included 24 females, seven from Lewa, 13 from Ol Jogi and four from Ol Pejeta. The Grevy's zebra breeding analysis has data points from three study reserves, both before and after the rainy period, so n=6.

Timing and spatial scale

Faecal samples were collected samples over two field seasons (Black rhino: June – July 2018 and January – March 2019; Grevy's: July-August 2018 and January-February 2019). These periods were chosen as they are after and before periods of expected rain respectively.
Samples were taken from eastern black rhino on Lewa, Ol Pejeta and Ol Jogi, and Grevy's zebra on Westgate, Ol Jogi, Mpala and. Zebra could move between the adjacent Ol Jogi, Mpala and Karisia areas so individuals from these areas were treated as one population for analysis. A map of the study reserves, and the distances between them can be seen in Supplementary Figure 7.

Data exclusions

We considered that a sample having under 1000 total reads for diet, and under 2000 for bacteria, after processing was indicative of sequencing failure, and removed those samples from further analysis. We removed 12 samples for black rhino diet, four samples for zebra diet, 27 samples for black rhino microbiome and three samples for zebra microbiome. This left us with the following sample sizes. Rhino diet: Lewa-67, Ol Jogi-97, Ol Pejeta-50. Rhino microbiome: Lewa-59, Ol Jogi-92, Ol Pejeta-48. Zebra diet: Lewa-45, Westgate-42:, Mpala-Ol Jogi (also includes Karisia samples)-67. Zebra microbiome: Lewa-45, Westgate-43:, Mpala-Ol Jogi (also includes Karisia samples)-67.

No other data were excluded.

Reproducibility

Whilst is difficult to apply strict principles of reproducibility to field-based ecological research, the sampling strategy was designed to give robust measures of differences across environmental gradients by sampling a range of different individuals, both male and female, right across each reserve and both pre- and post-rains. Grevy's zebra were not identified individually so there are no replicates for that species, but 38 black rhino individuals were sampled twice in the pre-rain period, and 29 post-rain. These repeat samples of the same black rhino individuals in the same sampling period show broadly similar dietary composition of the three study plant families although there is some variation. The diet and microbiome composition of study animals will vary naturally accordingly due to many factors, and so it is expected that sampling the same animal twice on different days will give different results even during the same season. Whilst there is intra-individual variation on each reserve, the clustering of each reserve on the principal component analyses (PCAs) and the consistent patterns of dietary and microbiome variation across these gradients and across many different individuals demonstrate reproducible effects of rainfall and NDVI.

Randomization

Randomisation is not relevant to this study. As we were studying Grevy's zebra and black rhino in different populations, study animals were separated into groups according to this. The different environmental conditions experienced by each study animal due to spatial separation were the dependent variables we were testing. Some environmental variables, such as soil type, are not included but the analysis focuses on seasonality.

Blinding

Although blinding is not that relevant to this study, all samples were assigned codes after they were selected for analysis. We used these codes to refer to each sample from DNA extraction through all the lab work and bioinformatic processing, so the researchers were not aware which individual animal or reserve each sample belonged to during this work. After bioinformatic processing, the species, reserve, season, individual, demographic information and environmental variables were reassigned to each sample code for analysis.

Did the study involve field work? ☒ Yes ☐ No

Field work, collection and transport

Field conditions

Woody plant communities in this ecosystem are dominated by species from the Fabaceae (including Vachellia and Senegalia species) and Ebenaceae (including Euclea divornum) families, and Poaceae (grasses). Ol Pejeta in Laikipia Country (0.02°N, 36.90°E) has an average annual rainfall of around 740 mm and the habitat cover types are dominated by grassland, V. drepanolobium wooded grassland and Euclea divinorum thicket 49,74. Ol Jogi also in Laikipia County (0.32°N, 36.98°) has an average annual rainfall of around

570 mm and is dominated by Vachellia and Senegalia woodland/thicket and has a smaller proportion of V. drepanolobium wooded grassland than the other two reserves 49. Lewa is sited in Meru County (0.20°N, 37.42°E), has an average annual rainfall of 570 mm and is dominated by V. drepanolobium wooded grassland with other habitats including mixed species bushland and mountain forest 49,75. Mpala is adjacent to Ol Jogi in Laikipia County (0.31°N, 36.96°E) and has annual rainfall of around 600 mm. It is dominated by V. drepanalobium bushland, Senegalia brevispica thicket and grassland 76. As Mpala and Ol Jogi are adjacent properties which Grevy's zebra, but not black rhino, can move freely between they were analysed as one area. Westgate is in Samburu County (0.81° N, 37.3°E), has an average annual rainfall of around 190 mm and is savanna grassland with varying densities of Vachellia, Commiphora (woody shrubs and trees in the Burseraceae family), Boscia (woody shrubs and trees in the Capparaceae family) and Grewia (woody shrubs and trees in the Malvaceae family)77. This region of Kenya traditionally has two annual rainy seasons; the long rains March-May and short rains October-December. Monthly rainfall peaks at around 100 mm in April and November on Lewa and Ol Pejeta, and around 80mm on Mpala and Ol Jogi. Mpala, Ol Jogi and Ol Pejeta have rainfall relatively well-spread throughout the rest of the year, with around 20-50 mm per month and a small peak in August, while Lewa has a pronounced dry period July-September49. Westgate has a similar timing of rains but lower amounts at all times of year compared to the other reserves. It should be noted that Kenya has been experienced a drought 2020-2023, but our samples were collected before this disruption to rainfall patterns.

Location

As above.

Access & import/export

Research was conducted in affiliation with the Kenya Wildlife Service and licensed by the Republic of Kenya's National Commission for Science & Innovation (Permit numbers: NACOSTI/P/17/87006/16178, NACOSTI/P/19/1947 and NACOSTI/P/19/310). No samples were exported from Kenya, analysis was carried out at ILRI in Nairobi.

Disturbance

The collection of dung sampling is non-invasive. We define non-invasive after Pauli et al. (2010) as a methodology where 'animals are unrestrained and do not exhibit a chronic or severe stress response or experience reduction in survival or reproduction'. Often, sampling was also unperceived where 'animals are unaware of sampling and, therefore, are unaffected by it' (Pauli et al. 2010). We were sometimes perceived by both rhino and zebra, but this was no different to the security patrols, ranger teams and tourist cars which also travel around the reserves. Sampling of dung that was observed being excreted was done after the animals had moved away.
We worked closely with the research, management and security teams on each reserve, tracking rhino and zebra with the help of rangers and monitoring teams. All fieldwork and sample collection was done with their guidance and help, and with the full knowledge of the Kenya Wildlife Service.

Reporting for specific materials, systems and methods

We require information from authors about some types of materials, experimental systems and methods used in many studies. Here, indicate whether each material, system or method listed is relevant to your study. If you are not sure if a list item applies to your research, read the appropriate section before selecting a response.

Materials & experimental systems

n/a	Involved in the study
☒	☐ Antibodies
☒	☐ Eukaryotic cell lines
☒	☐ Palaeontology and archaeology
☐	☒ Animals and other organisms
☒	☐ Clinical data
☒	☐ Dual use research of concern
☒	☐ Plants

Methods

n/a	Involved in the study
☒	☐ ChIP-seq
☒	☐ Flow cytometry
☒	☐ MRI-based neuroimaging

Animals and other research organisms

Policy information about studies involving animals; ARRIVE guidelines recommended for reporting animal research, and Sex and Gender in Research

Laboratory animals

Study did not involve laboratory animals.

Wild animals

We took dung samples from eastern black rhino (Diceros bicornis michaeli) across three Kenyan reserves, Lewa, Ol Jogi and Ol Pejeta, and from Grevy's zebra (Equus grevyi) across three Kenyan conservancies and a research centre. All sampling was non-invasive. We worked closely with the research, management and security teams on each reserve and travelled around the reserves either with a ranger in the car or maintaining constant communication with the control centre.
For black rhino, all samples came from known individuals. This was possible due to the monitoring carried out on each reserve. These were either collected from dung that we observed being excreted, after waiting for the individual to move away, or from fresh dung in middens that the monitoring teams informed us could be certainly attributed to a particular individual. Samples were collected from adults, sub-adults and calves, but only samples from adults are used in this study.
For Grevy's zebra, all samples were collected from dung that we observed being excreted, after waiting for the individual to move away. Individuals were sexed by sight. Samples were collected from adults, sub-adults and calves, but only samples from adults are used in this study.

Reporting on sex

Black rhino and Grevy's zebra samples were taken from both males and females. On the study reserves, black rhino individuals are known by the monitoring and ranger teams, either by ear notches, horn shape or other identifying characteristics. As we only took samples from known individuals, the sex of the individual that each sample came from was known. 103 samples came from females and 123 from males.
For Grevy's zebra, individuals that samples came from were sexed by sight. The black part of the rump of a female, under the tail is much broader and longer on a mare than on a stallion. This dark part is where the anus and the vulva of the mare are located. 79 samples came from females and 79 from males.
The only analysis which includes only one sex is the rhino breeding analysis, which only includes females. Paternity is difficult to assign without the use of genetic techniques, as mating is not always observed and females may mate with several males during oestrus. Due to this uncertainty, we did not include data on male breeding in the analyses. We tested whether sex was an important predictor in all other analyses and it was found not to be, so we do not include results disaggregated by sex.

Field-collected samples

For black rhino, we collected samples from at least two complete boluses per dung pile, from several areas of each bolus and avoiding the surface 1cm depth. For Grevy's zebra, several whole droppings were taken from each dung pile, avoiding those touching soil of vegetation. During a sampling session, samples were placed in unused sealed plastic bags and had excess air removed. These were placed in a cool bag or box in the car. Within 6 hours from defecation, usually within 4 hours, we homogenised samples in their sample bags, and then removed around 3g using sterile implements. We stored samples in 8ml of 100% ethanol during the field season up to a maximum of nine weeks. Storage in ethanol has been shown to be effective for the extraction of DNA up to six months after sample collection. These tubes were then stored at -20°C until DNA extraction in November 2019.

Ethics oversight

This project was approved by the University of Manchester's Committee for the ethical review of category D research (Ref: 0030). Research was conducted in affiliation with the Kenya Wildlife Service and licensed by the Republic of Kenya's National Commission for Science & Innovation (Permit numbers: NACOSTI/P/17/87006/16178, NACOSTI/P/19/1947 and NACOSTI/P/19/310).

Note that full information on the approval of the study protocol must also be provided in the manuscript.
